# Rescue of a duck circovirus from an infectious DNA clone in ducklings

**DOI:** 10.1186/s12985-015-0312-6

**Published:** 2015-05-30

**Authors:** Pengfei Li, Zhilong Zhang, Renyong Jia, Sai Mao, Mingshu Wang, Ruiling Jia, Mafeng Liu, Dekang Zhu, Shun Chen, Kunfeng Sun, Zhongqiong Yin, Xiaoyue Chen, Anchun Cheng

**Affiliations:** Institute of Preventive Veterinary Medicine, Sichuan Agricultural University, Chengdu, People’s Republic of China; Avian Disease Research Center, Sichuan Agricultural University, Ya’an, People’s Republic of China; Key Laboratory of Animal Disease and Human Health of Sichuan Province, Chengdu, People’s Republic of China

**Keywords:** Duck circovirus, Infectious clone, Virus rescue

## Abstract

**Background:**

Duck circovirus may predispose the host to immunosuppression and may serve as an immunological trigger for further complicated disease progression. Due to the lack of a cell culture system for propagating DuCV, little is known regarding the molecular biology and pathogenesis of DuCV. The aim of this study was to describe the construction and initial *in vivo* characterization of full-length DNA clones of DuCV (pIC-Mu2DuCV) and its infectivity under *in vivo* conditions.

**Method:**

The constructed pIC-Mu2DuCV contained two copies of the whole DuCV genome and an introduced *Xho* I restriction enzyme site. Eighty-one 10-day-old conventional ducklings that were free of DuCV were randomly divided equally into three groups (1, 2 and 3). The ducklings in groups 1, 2 and 3 were inoculated intramuscularly with pIC-Mu2DuCV, wild-type virus GH01 and PBS, respectively. Subsequently, all of the ducklings were examined clinically, which were each given a physical condition score, and their rectal temperatures were taken daily during the experimental period. DuCV genomes in serum samples and in various tissues from all of the ducklings at 0, 1, 3, 5, 7, 10, 15, 21 and 28 DPC were detected by PCR and real-time quantitative PCR, respectively.

**Results:**

The average daily weight gain (ADWG) of group 3 was significantly higher than those of groups 1 and 2, and the temperature of all ducklings was stable between 41.7 °C and 42.2 °C. The clinical values (physical condition scores) of groups 1, 2 and 3 were 12.5, 15.6 and 0, respectively. In addition, viremia occurred at 15 and 10 days post-challenge (DPC) in groups 1 and 2, and antibodies could be detected in these ducklings at 21 and 15 DPC. Proliferation ability analysis showed that the viral titers of group 1 were lower than those of their parental viruses in group 2.

**Conclusion:**

This study shows that the rescued viruses are not significantly different but exhibit lower pathogenicity and proliferation ability compared with the parental virus. The results will facilitate future studies on DuCV pathogenesis and biology.

## Introduction

The duck circovirus (DuCV) is a member of the genus *Circovirus* within the family *Circoviridae*. The DuCV virion is icosahedral, non-enveloped, and 15 to 16 nm in diameter [1]. DuCV was originally reported in two female 6-week-old Mulard ducks from a German farm; both ducks had a feathering disorder and poor body condition [1, 2]. The virus has since been reported in Hungary [3], Taiwan [4], the US [5] and Mainland China [6–9]. DuCV has been detected in Muscovy, Mule and Pekin ducks, where it causes stunting and feather abnormalities. Although controversial, lymphoid depletion predisposes the host to immunosuppression, and disease progression is further complicated by co-infections with other bacterial and viral pathogens [2, 10].

DuCV consists of a single-stranded, circular DNA genome that contains approximately 1988–1996 nucleotides (nts) and two major open reading frames (ORFs) [1]. ORF1, denoted the *rep* gene, encodes the replication-associated protein, which is required for viral replication initiation. Meanwhile, ORF2, denoted the *cap* gene, encodes a viral structural and virulence-associated protein that stimulates the host immune response. The intergenic regions of these ORFs contain a stem loop, which is considered the site of viral DNA replication initiation [1, 5].

At present, DuCV is not considered to be directly associated with a particular disease, although recent studies have suggested that DuCV partially contributes to lymphoid depletion [2], may predispose the host to immunosuppression and may serve as an immunological trigger for further complicated disease progression [3–7]. Indeed, DuCV-affected ducks exhibited a higher prevalence and greater loads of other bacterial and viral pathogens than non-DuCV-affected ducks [1, 11]. However, the results from the above-mentioned studies do not support a direct association of DuCV with another pathogen or with host damage.

Due to the lack of a cell culture system for propagating DuCV, little is known regarding the molecular biology and pathogenesis of DuCV. To definitively characterize diseases associated with DuCV infection, an appropriate animal model is needed [12]. In addition, reverse genetics is a powerful tool for addressing these questions [13–15]. Because infections with multiple different genotypes or subtypes of DuCV are common events, a biologically pure and isolated form of a specific DuCV that is generated from a full-length infectious DNA clone is also required to study the pathology due to a single phenotype [15]. To date, no infectious DNA clones of DuCV in cultured cells or animals have been reported; therefore, it is important to construct an infectious DuCV DNA clone that can be used as a model for studying the replication and transcription mechanisms of DuCV as well as for dissecting the structural and functional relationships between host and DuCV genes. Here, we describe the construction and initial *in vivo* characterization of full-length DNA clones of DuCV. Furthermore, the rescue of a DuCV containing the introduced genetic markers was confirmed by sequencing of viral DNA obtained from ducks experimentally inoculated with circular DuCV genomic DNA.

## Materials and methods

### Ethics statement

The experimental procedures were performed in strict accordance with the *Guidance Suggestions for the Care and Use of Laboratory Animals* and were approved by the National Institute of Animal Health Animal Care and Use Committee of Sichuan Agricultural University (Approval Number 2012–032).

### Viruses and animals

Duck circovirus strain GH01 (GenBank No. JX499186) was isolated and maintained at the Institute of Preventive Veterinary Medicine of Sichuan Agricultural University. A cloned strain with a genetic marker, termed RMDV, was obtained and used as the animal-challenge strain in this study to avoid contamination by the parental virus and other unknown viruses. Ninety-six healthy, 10-day-old commercial ducklings were obtained from a duck farm that was negative for DuCV, as detected by PCR.

### Construction of a DuCV molecular DNA clone

**Fig. 1:**
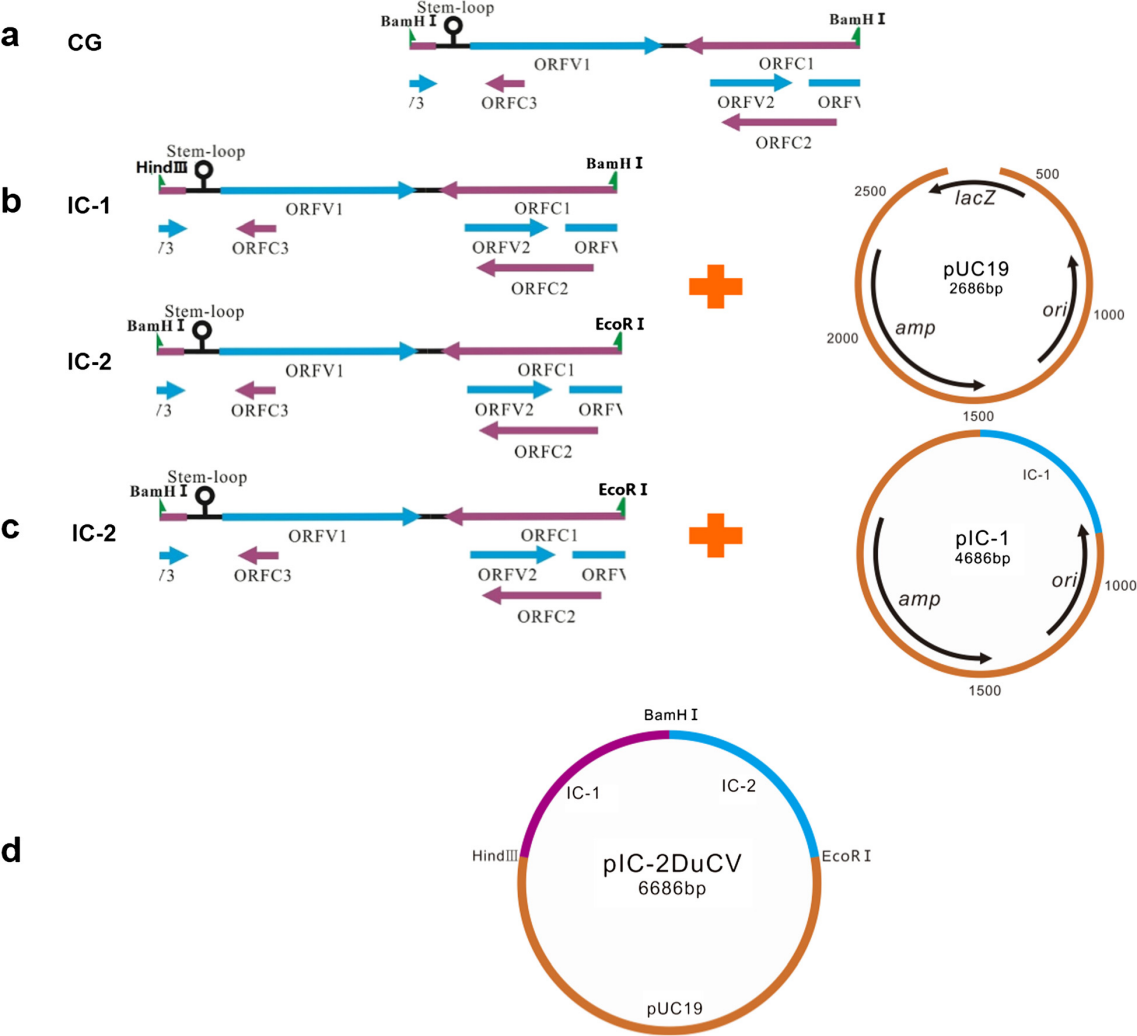
Construction strategy of pIC-2DuCV. **a** Two full-length genomes of DuCV strain GH01, denoted IC1 and IC2, were amplified. **b** IC1 and IC2 were ligated into the pUC19 vector to yield pIC-1 and pIC-2, respectively. **c** IC-2 was ligated head-to-tail to pIC-1 to produce a tandem-dimerized DuCV DNA clone, which was denoted pIC-2DuCV. **d** pIC-2DuCV

The full-length genome of DuCV strain GH01 was amplified by PCR using two pairs of primers, namely IC-1F/IC-1R and IC-2 F/IC-2R (Fig. 1a), and the amplification products were named IC1 and IC2, respectively. The products were subsequently inserted into a pUC19 vector (TaKaRa, Dalian, China) that had been previously digested with *Hind* III/*BamH* I or *BamH* I/*EcoR* I, respectively. The resulting constructs were termed monomeric DuCV DNA pIC-1 or pIC-2, respectively, and then transformed into *Escherichia coli* DH5α competent cells (Fig. 1b). The recombinant plasmids were verified by PCR, restriction enzyme digestion and DNA sequencing. The full-length IC-2 was excised from pIC-2 by digestion with the *BamH* I and *EcoR* I restriction enzymes, gel-purified and ligated head-to-tail with pIC-1 to construct a tandem-dimerized DuCV DNA clone, which was denoted pIC-2DuCV (Fig. 1c and 1d). pIC-2DuCV was also confirmed by PCR, restriction enzyme digestion and DNA sequencing.

### Introduction of genetic markers into the tandem-dimerized DuCV DNA clone

**Fig. 2:**
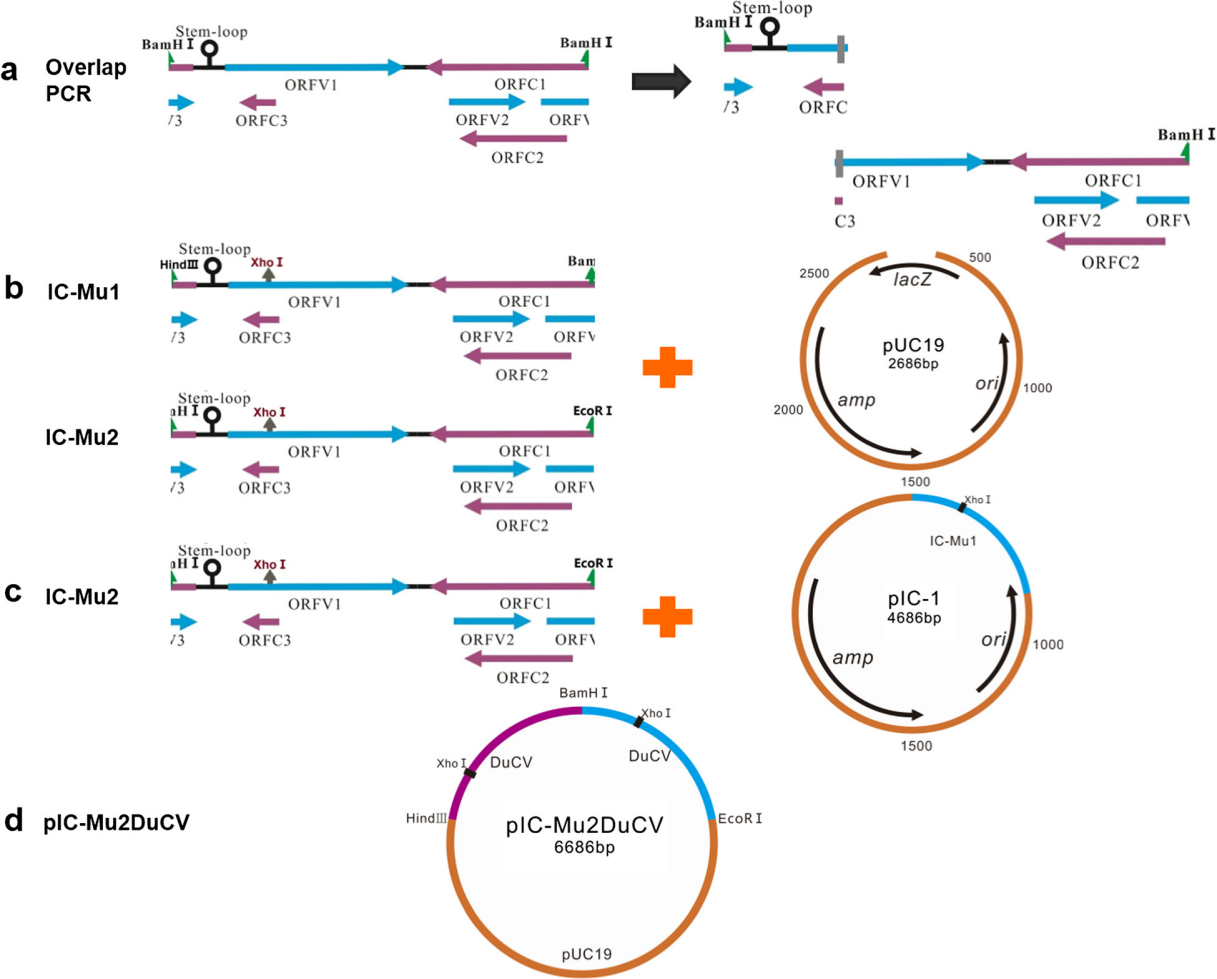
Construction strategy of pIC-Mu2DuCV. **a** Two full-length genomes of DuCV strain GH01, denoted IC-Mu1 and IC-Mu2, were amplified by overlapping PCR. **b** IC-Mu1 and IC-Mu2 were ligated into the pUC19 vector to yield pIC-Mu1 and pIC-Mu2, respectively. **c** IC-Mu2 was ligated head-to-tail to pIC-Mu1 to produce a tandem-dimerized DuCV DNA clone, which was denoted pIC-Mu2DuCV. **d** pIC-Mu2DuCV

An *Xho* I restriction enzyme site was engineered into the DuCV genome within the pIC-2DuCV clone to introduce a genetic marker that would allow discrimination between the cloned virus and the potential indigenous viruses in the subsequent animal study. To create the unique *Xho* I site (C′TCGAG; mutation is underlined), an A-to-G point mutation at nucleotide position 482 of the IC-2 genome was generated by a fusion PCR technique using two pairs of primers (IC-MuF/IC-R1 and IC-F1/IC-MuR) containing the desired mutations (Fig. 2a). The corresponding region in GH01 was replaced by the fusion PCR product using the *Xho* I sites at both ends. The mutation did not change the putative ORFV1 or its complementary amino acid sequence. The resulting full-length DNA clones were named IC-Mu1 and IC-Mu2, respectively (Fig. 2b). All mutations were confirmed by restriction enzyme digestion and DNA sequencing. Using the same strategy, IC-Mu1 and IC-Mu2 were ligated to the pUC19 vector to produce a tandem-dimerized DuCV DNA clone, denoted pIC-Mu2DuCV (Fig. 2c and 2d).

### *In vivo* transfection of ducklings with the DuCV molecular DNA clone

To determine whether the plasmid DNA of the dimerized DuCV or muDuCV clone was infectious when directly injected intramuscularly into ducklings, fifteen 10-day-old ducklings were randomly assigned into three rooms of five animals each, and prior to inoculation, the absence of DuCV in the ducklings was verified by PCR. The ducklings were then inoculated intramuscularly with approximately 100 μg/kg recombinant plasmid DNA pIC-2DuCV, pIC-Mu2DuCV or pUC19 vector combined with 200 μg/kg Lipofectamine 2000 (Invitrogen, Shanghai) (Table 1). Blood samples were then collected at 0 (before inoculation), 1, 3, 7, 10, 15, 21 and 28 DPC and submitted to DuCV detection.

**Table 1:** The grouping and dosage of transfection experiment

Groups	Quantity	Inoculation route	Date of inoculation	Inoculation dosage		
				DNA(μg/kg)	Lipofectamine(μg/kg)	pUC19(μg/kg)
A	5	i.m.	10-day-old	100	200	-
B	5	i.m.	10-day-old	100	200	-
C	5	i.m.	10-day-old	-	200	100

Eighty-one 10-day-old ducklings were divided randomly into two challenge groups (groups 1 and 2) and a control group (group 3; 27 ducklings in each group) and were raised separately in different isolation rooms with individual ventilation systems. The animals received food and water ad libitum. The group-1 and group-2 ducklings were inoculated intramuscularly with a defined dose (0.5 ml × 105 ID50/ml) of pIC-Mu2DuCV, and the ducklings in the control group were mock-inoculated with the same volume of PBS using the above-mentioned inoculation route and procedure (Table 2). On the day of challenge and at the end of the experiment, all of the ducklings were weighed, and the average daily weight gain (ADWG) (kg/day) was determined and expressed as (body weight at 28 DPC – body weight at 0 DPC)/28. Blood samples were obtained at 0, 1, 3, 5, 7, 10, 15, 21 and 28 DPC for virological and serological examination. After challenge, all of the ducklings were examined clinically, and their rectal temperatures were taken daily during the experimental period. At each examination point, three randomly selected ducklings were killed and subjected to postmortem examinations, determination of any gross lesions, and pathological examinations.

**Table 2:** The grouping and dosage of transfection experiment

Groups	Quantity	Inoculation route	Date of inoculation	Inoculum and dosage		
				pIC-Mu2DuCV	WT-DuCV	PBS
1	27	i.m.	10-day-old	100 μg/kg	-	-
2	27	i.m.	10-day-old		0.5 ml × 10^5^ID_50_/ml	-
3	27	i.m.	10-day-old			0.5 ml

### Clinical examination

The ducklings of the above-mentioned DuCV-challenged groups and of the control group were examined clinically at 0, 1, 3, 5, 7, 10, 15, 21 and 28 DPC and were each given a physical condition score. The evaluated clinical signs included the presence of wasting, feathering disorder and depression. The clinical parameters were scored using a numeric value ranging from 0 to 2 (0 = normal, 1 = mild, 2 = severe).

### Serology for DuCV antibody detection

The presence of specific antibodies against DuCV in serum samples obtained throughout the study at 0, 1, 3, 5, 7, 10, 15, 21 and 28 DPC was determined by ELISA using a protocol established by our laboratory (data not published). ELISA was also used to detect changes in the DuCV-specific antibodies over time *in vivo* post DuCV challenge. Ninety-six-well ELISA plates (Corning, USA) were coated with optimized prepared Cap peptide solution (data not published) and incubated at 4 °C overnight. After washing three times with PBS containing 0.05 % Tween-20 (PBST), the plates were blocked with 1 % bovine serum albumin (BSA) in PBS for 1 h at 37 °C. Following this incubation, the wells were washed three times with PBST, duck serum diluted with 1 % BSA-PBST (1:50) was added, and the plates were incubated at 37 °C for 1 h. After three washes, as described above, HRP-labeled goat anti-duck IgG (1:5000) was added, and the plates were incubated at 37 °C for 1 h. After three additional washes, 100 μL of TMB was added to each well, and the plates were incubated at 37 °C for 20 min for color development. The color-development reaction was terminated by adding 100 μL of 2 M H_2_SO_4_ to each well, and the absorbance of each well at a wavelength of 450 nm (OD_450 nm_) was determined using a microplate reader (Bio-Rad).

### Detection of DuCV viremia in serum samples by PCR

Serum samples were collected from all of the ducklings at 0, 1, 3, 5, 7, 10, 15, 21 and 28 DPC, and DuCV nucleic acids were detected using the primer pair PD-F/PD-R by PCR. Briefly, PD-F and PD-R were used to amplify a 774-bp-long product, and viral DNA was extracted from the positive serum samples using a DNA extraction kit (Tiangen, Beijing) according to the manufacturer’s instructions. The amplification was performed in a 20-μL reaction mixture containing 10 μL of DNA polymerase mix (Tiangen, Beijing), 0.4 mM of each primer, 0.4 μL of extracted DNA and 8.8 μL of H_2_O and using the following cycling program: 2 min at 94 °C; 35 cycles of 30 s at 94 °C, 30 s at 62 °C and 30 s at 72 °C; and a final extension step at 72 °C for 5 min. To differentiate the rescued virus from the parental virus, restriction enzyme digestion and DNA sequencing were performed.

### Real-time quantitative PCR (qPCR) to detect DuCV nucleic acids in different tissues

Three experimental ducklings from each group were sacrificed at 0, 1, 3, 5, 7, 10, 15, 21 and 28 DPC, and various tissues were collected for DuCV nucleic acid extraction using a DNA extraction kit (Tiangen, Beijing) according to the manufacturer’s instructions. To quantify the number of DuCV genomes in the samples, a qPCR technique established in this laboratory (data not published), using the SYBR-F (5′-ACTGACGTTGCCCGGAAGTA-3′, position 426-445) and SYBR-F (5′-TCTTCCAATCACGTTGCGTTT-3′, position 505-485) primer sequences, were used (Table 3). Amplification was performed in a 20-μL reaction mixture containing 10 μL of iTaq™ Universal SYBR® Green (Bio-Rad, Beijing), 0.4 mM of each primer, 0.4 μL of extracted DNA and 8.8 μL of double-distilled H_2_O. The thermal profile for the SYBR Green PCR was 95 °C for 30 s, followed by 39 cycles of 95 °C for 10 s and 60 °C for 15 s. The results of the qPCR are expressed as the logarithm of the copies of the DuCV genome per gram of sample (log copies/gram).

**Table 3:** Nucleotide sequences of the primer

Primers^a^	Sequences (5′-3′)^b^	Usage
IC-1F	CCC*AAGCTT*GGAACTGGACCAAC	Fragment clone
IC-1R	TCC*GGATCC*GAAAAATCCAAATAC	
IC-2F	TTC*GGATCC*GGAACTGGACCAAC	Fragment clone
IC-2R	TCC*GAATTC*GAAAAATCCAAATACGG	
IC-MuF	GATCAGGTGACGAAGGCG*CTCGAG*GCCACGCCCAAAG	Genetic marker
IC-MuR	CTTTGGGCGTGGC*CTCGAG*CGCCTTCGTCACCTGATC	
PD-F	TGAACCCGGTGAACTGACC	Genetic marker detection
PD-R	ATGCGACGCAGCACCTATC	
SYBR-F	ACTGACGTTGCCCGGAAGTA	Quantitate virus stock
SYBR-R	TCTTCCAATCACGTTGCGTTT	

^a^F denotes forward PCR primer; R denotes reverse transcription or reverse PCR primer

^b^Italics and underlines stand for restriction enzyme site

### Statistical analysis

Comparisons of a single treatment among the different challenge groups (using the ADWG, ELISA and qPCR results) were all performed using nonparametric one-way ANOVA followed by LSD multiple comparisons. The statistical analysis of the data was performed using SPSS for Windows (version 16.0), and P < 0.05 was considered statistically significant in all cases.

## Results

### Construction of an infectious viral clone

**Fig. 3:**
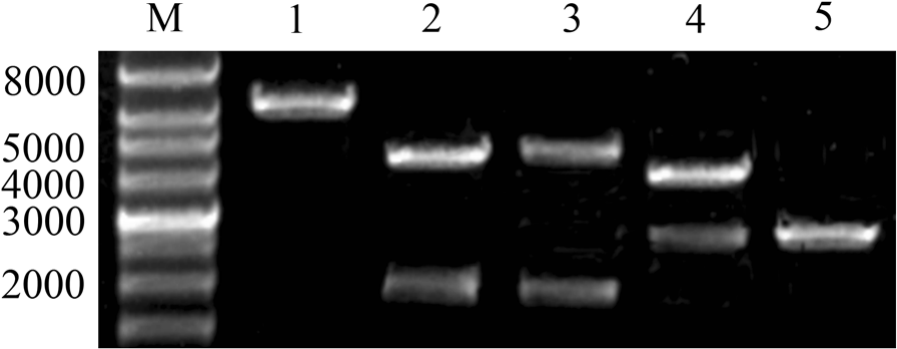
Identification of the recombinant plasmid pIC-2DuCV by restriction enzyme digestion. M, wide-range DNA marker (500 ~ 12,000); 1, digested with *Hind* III; 2, digested with *Hind* III and *BamH* I; 3, digested with *BamH* I and *EcoR* I; 4, digested with *Hind* III and *EcoR* I; 5, pUC19 digested with *Hind* III

We first generated two monomeric, full-length DuCV DNA clones, denoted IC-1 and IC-2, that were derived from the prototype Sichuan isolate GH01 (Fig. 1a and 1b). Each full-length DuCV genome was inserted into a pUC19 vector containing a eukaryotic promoter. *Hind* III/*BamH* I and *BamH* I/*EcoR* I, which were unique restriction sites in the pIC-1 and pIC-2 genomes, respectively, were incorporated at the ends of the genomic DNA to facilitate the generation of concatemers and thus mimic the DuCV circular DNA genome. Double digestion of the plasmid DNA of each clone with *BamH* I or *EcoR* I resulted in 2.6-kb, 2-kb, and 4.6-kb fragments. The 2.6-kb fragment represented the backbone vector, whereas the 2-kb fragment represented the inserted monomeric DuCV genomic DNA. The 4.6-kb fragment consisted of the linearized backbone vector with the inserted monomeric DuCV genomic DNA. Subsequently, the 4.6-kb and 2-kb fragments were ligated in tandem to generate the pIC-2DuCV clone, and the recombinant plasmids were verified by PCR, restriction enzyme digestion and DNA sequencing (Fig. 3).

**Fig. 4:**
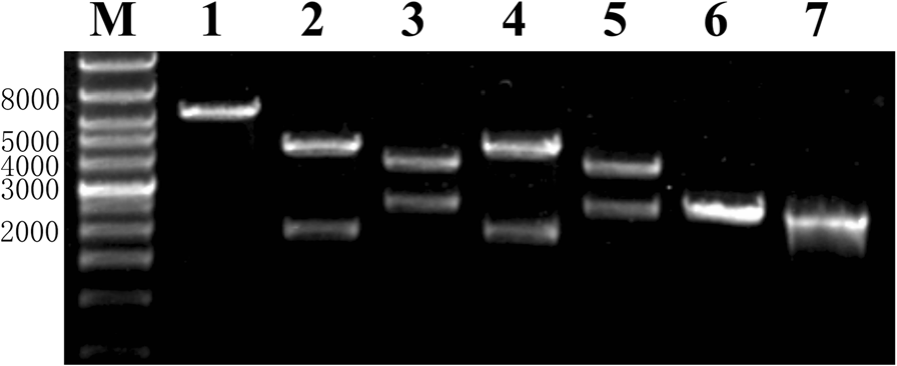
Identification of recombinant pIC-Mu2DuCV plasmid by restriction enzyme digestion. M, wide-range DNA marker (500 ~ 12,000); 1, digested with *Hind* III; 2, digested with *Hind* III and *BamH* I; 3, digested with *BamH* I and *EcoR* I; 4, digested with *Hind* III and *EcoR* I; 5, digested with *Xho* I; 6, pUC19 digested with *Hind* III; 7, pUC19

A fusion PCR technique using two pairs of primers (IC-MuF/IC-R1 and IC-F1/IC-MuR) containing the desired mutations was utilized to create the unique *Xho* I site (Fig. 2a). The resulting fusion PCR products were named IC-Mu1 and IC-Mu2, respectively (Fig. 2a and 2b), and processing was continued using the above-described technique to produce a tandem-dimerized DuCV DNA clone, which was denoted pIC-Mu2DuCV (Fig. 2c and 2d). All mutagenesis was confirmed by restriction enzyme digestion and DNA sequencing (Fig. 4).

### The tandem-dimerized DuCV clones pIC-2DuCV and pIC-Mu2DuCV are infectious when inoculated into ducklings

**Fig. 5:**
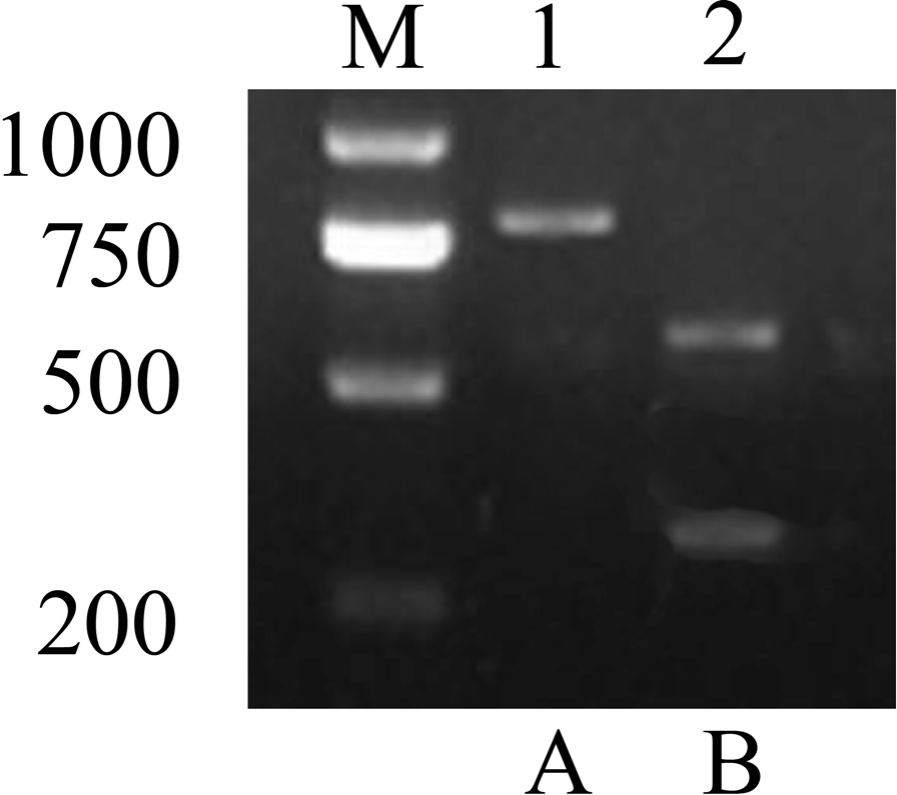
Identification of DNA from groups A and B. M, DNA marker; 1, **a** digested with *Xho* I; 2, **b** digested with *Xho* I

The ducklings were inoculated intramuscularly with approximately 100 μg/kg of pIC-2DuCV or pIC-Mu2DuCV recombinant plasmid DNA or pUC19 vector combined with 200 μg/kg Lipofectamine 2000 (Table 1). Blood samples were then collected at 0 (before inoculation), 1, 3, 7, 10, 15, 21 and 28 DPC and used for DuCV detection. The viral nucleic acids in the serum in the challenged groups were detected at 15 DPC using PCR (Table 4), and restriction enzyme digestion and DNA sequencing were performed to differentiate the rescued virus from the parental virus (Fig. 5). The results from these experiments indicated that the constructed full-length cDNA clones were infectious and that viable viruses could be recovered; the rescued virus containing the genetic marker was named RMDV.

**Table 4:** The results of transfection experiment

Groups	DPC								
	0	1	3	5	7	10	15	21	28
A	0/5^a^	0/5	0/5	0/5	0/5	0/5	2/5	4/5	5/5
B	0/5	0/5	0/5	0/5	0/5	0/5	2/5	5/5	5/5
C	0/5	0/5	0/5	0/5	0/5	0/5	0/5	0/5	0/5

a:Number of DuCV positive/Number of ducklings tested

Because the genetic marker that was introduced into the full-length DNA clones can be used to distinguish between infections caused by the cloned virus or by a potential indigenous contaminating virus, we performed a study to further verify the *in vivo* infectivity of the RMDV rescue mutant virus in ducklings. Eighty-one ducklings were assigned to three groups of 27 ducklings each, and the ducklings in each group were inoculated with RMDV, WT-DuCV or PBS.

### Clinical examination

No apparent gross lesions were observed in the control group, but clinical signs characterized by wasting, feathering disorder and depression were observed mainly from 10 to 28 DPC. The mean clinical score (CS) was significantly higher (P < 0.05) in the RMDV- and WT-DuCV-challenged groups compared with the PBS-challenged group throughout the study period, but no significant differences were observed between the RMDV- and WT-DuCV-challenged groups (P > 0.05). The detailed CS values are shown in Table 5.

**Table 5:** Scored values for clinical condition for ducklings in different challenge groups and the control group

Sign	RMDC	WT-DuCV	PBS
Wasting	13 × 0,12 × 1,2 × 2^a^	10 × 0,14 × 1,3 × 2	27 × 0,0 × 1,0 × 2
Feathering disorder	18 × 0,9 × 1,0 × 2	16 × 0,9 × 1,1 × 2	27 × 0,0 × 1,0 × 2
Depression	15 × 0,11 × 1,1 × 2	13 × 0,12 × 1,2 × 2	27 × 0,0 × 1,0 × 2
Total median score	12.6^b^	15.6*	0

^a^The clinical parameters were scored using a numeric value ranging from 0 to 2 (0 = normal, 1 = mild, 2 = severe); number of elements × scores

^b^Pairs of treatments with (*) were significantly different with control group (p < 0.05)

**Fig. 6:**
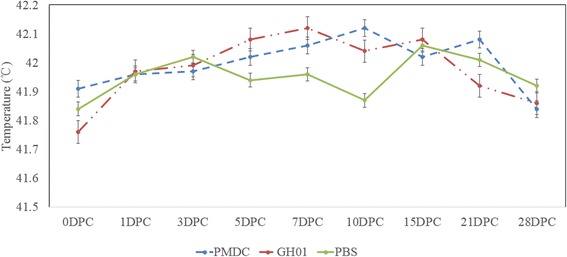
Changes in the rectal temperatures of the ducklings from the different experimental groups. The data are presented as the means ± SD

The rectal temperatures of the ducklings in both of the challenge groups and in the control group did not exceed 42.2 °C up to the termination of the experiment (Fig. 6).

**Fig. 7:**
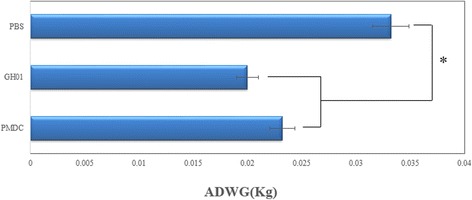
Comparison of the average daily weight gains for the different experimental groups. *indicates a statistically significant difference (P < 0.05)

As shown in Fig. 7, the ADWG was higher in the control group than in the challenge groups, and this difference was statistically significant (P < 0.05). Moreover, no significant difference in ADWG was observed between the RMDV-challenged group and the WT-DuCV-challenged group (P > 0.05), which could represent persuasive proof of the similar virulence of RMDV and WT-DuCV. The results confirmed similar virulence of RMDC as WT-DuCV in ducklings, but further demonstrations are required.

### Viremia

To detect viremia, the viral DNA from serum samples was collected at 0, 1, 3, 5, 7, 10, 15, 21 and 28 DPC and investigated using routine PCR. Moreover, restriction enzyme digestion and DNA sequencing were performed to differentiate the rescued virus from the parental virus (data not shown). No viremia was detected in the ducklings of the control group, whereas ducklings challenged with RMDV showed viremia from 15 to 28 DPC. In addition, earlier and more severe viremia was observed in the WT-DuCV-challenged group compared with the RMDV-challenged group (Table 6).

**Table 6:** Examinations of DuCV in sera of ducklings challenged experimentally with different DuCV strains at different time-points post challenge with PCR

Groups	DPC								
	0	1	3	5	7	10	15	21	28
RMDV	0/27^a^	0/24	0/21	0/18	0/15	0/12	4/9	5/6	3/3
WT-DuCV	0/27	0/24	0/21	0/18	0/15	3/12	7/9	6/6	3/3
PBS	0/27	0/24	0/21	0/18	0/15	0/12	0/9	0/6	0/3

^a^Number of DuCV positive/Number of ducklings tested

### Detection of DuCV-specific antibody

**Fig. 8:**
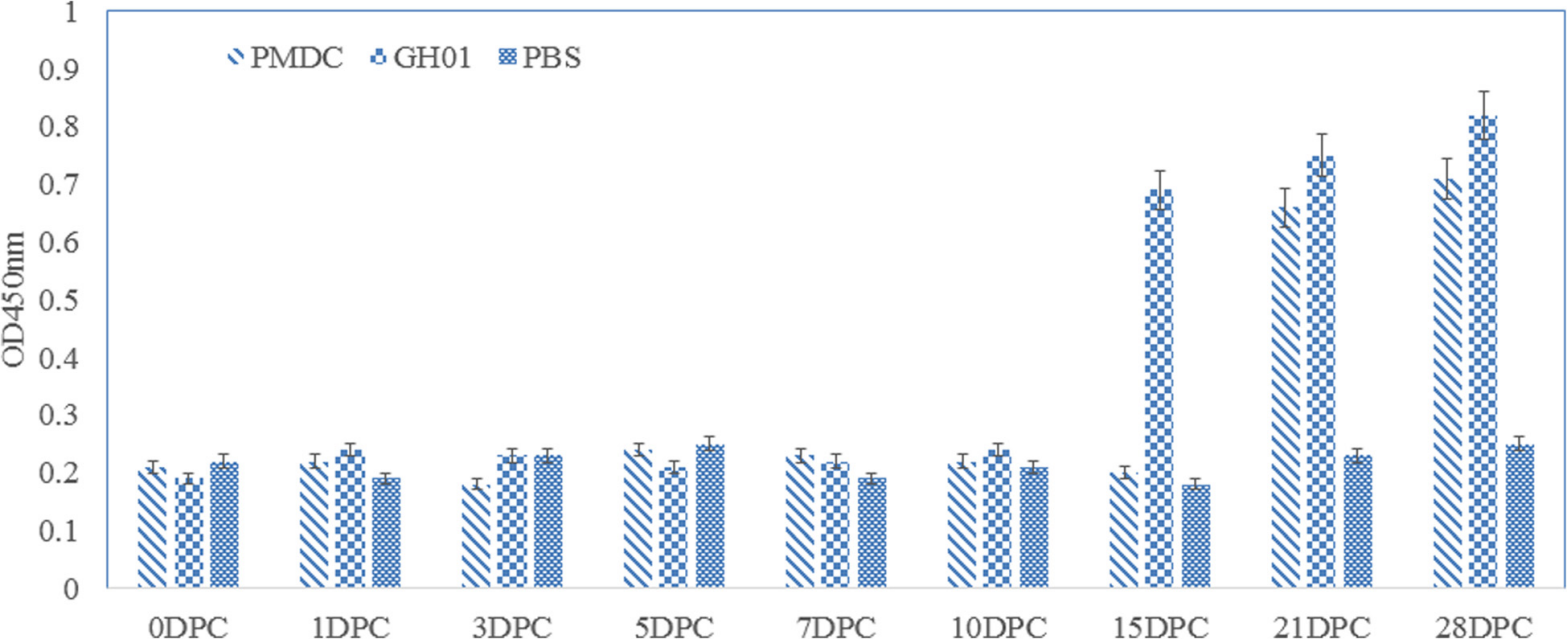
Detection of DuCV-specific antibodies in sera at different time points

ELISAs were used to investigate the level of DuCV-specific antibodies in the serum of ducklings from the DuCV-challenged and control groups. As shown in Fig. 8, DuCV-specific antibodies appeared at approximately 21 DPC in RMDV-challenged ducklings and at approximately 15 DPC in WT-DuCV-challenged ducklings, and the antibody titers increased slightly. In contrast, no DuCV-specific antibodies were detected in the control-group ducklings.

### Virus distribution and quantification in different tissues, as shown by real-time quantitative PCR (qPCR)

DuCV was localized mainly in the bursa of Fabricius (BF), spleen, liver, kidney, thymus, and Harderian gland of the ducklings. The earliest time point at which virus was observed in the serum of RMDV-challenged ducklings was 15 DPC, which was 5 days later than the earliest time point at which virus was observed in WT-DuCV-challenged ducklings; however, the viral loads of RMDV-challenged ducklings at 15 DPC were lower than 10^4^ copies/mg. Meanwhile, the viral loads in the BF of all of the challenged groups were significantly greater than those of the other tissues (P < 0.05) and exceeded 10^4^ copies/mg or 10^5^ copies/mg, reaching the highest load at 21 DPC (4.72 × 10^4^–8.34 × 10^4^ copies/mg in the RMDV-challenged group and 3.25 × 10^5^ ~ 5.47 × 10^5^ copies/mg in the WT-DuCV-challenged group). In addition, no virus was detected in the control group. The tissue sample qPCR results are summarized in Table 7.

**Table 7:** Quantification and distribution of viral DNA loads in the different experimental ducklings

DPC	NO.	PMDC							GH01							PBS						
		BF	Sp	Li	K	T	Hg	Se^b^	BF	Sp	Li	K	T	Hg	Se	BF	Sp	Li	K	T	Hg	Se
0	1	-^a^	-	-	-	-	-	-	-	-	-	-	-	-	-	-	-	-	-	-	-	-
	2	-	-	-	-	-	-	-	-	-	-	-	-	-	-	-	-	-	-	-	-	-
	3	-	-	-	-	-	-	-	-	-	-	-	-	-	-	-	-	-	-	-	-	-
1	4	-	-	-	-	-	-	-	-	-	-	-	-	-	-	-	-	-	-	-	-	-
	5	-	-	-	-	-	-	-	-	-	-	-	-	-	-	-	-	-	-	-	-	-
	6	-	-	-	-	-	-	-	-	-	-	-	-	-	-	-	-	-	-	-	-	-
3	7	-	-	-	-	-	-	-	-	-	-	-	-	-	-	-	-	-	-	-	-	-
	8	-	-	-	-	-	-	-	-	-	-	-	-	-	-	-	-	-	-	-	-	-
	9	-	-	-	-	-	-	-	-	-	-	-	-	-	-	-	-	-	-	-	-	-
5	10	-	-	-	-	-	-	-	-	-	-	-	-	-	-	-	-	-	-	-	-	-
	11	-	-	-	-	-	-	-	-	-	-	-	-	-	-	-	-	-	-	-	-	-
	12	-	-	-	-	-	-	-	-	-	-	-	-	-	-	-	-	-	-	-	-	-
7	13	-	-	-	-	-	-	-	-	-	-	-	-	-	-	-	-	-	-	-	-	-
	14	-	-	-	-	-	-	-	-	-	-	-	-	-	-	-	-	-	-	-	-	-
	15	-	-	-	-	-	-	-	-	-	-	-	-	-	-	-	-	-	-	-	-	-
10	16	-	-	-	-	-	-	-	+	-	-	-	-	-	+	-	-	-	-	-	-	-
	17	-	-	-	-	-	-	-	+	-	-	-	+	-	+	-	-	-	-	-	-	-
	18	-	-	-	-	-	-	-	-	-	-	-	-	-	-	-	-	-	-	-	-	-
15	19	+	-	+	-	-	-	+	++	-	+	+	-	-	+	-	-	-	-	-	-	-
	20	+	+	-	-	-	-	+	++	+	+	+	-	-	+	-	-	-	-	-	-	-
	21	-	-	-	-	-	-	-	+	+	-	-	+	-	+	-	-	-	-	-	-	-
21	22	++	+	+	+	+	+	+	+	-	+	+	-	-	+	-	-	-	-	-	-	-
	23	+	-	+	+	-	-	+	+++	+	++	+	+	+	+	-	-	-	-	-	-	-
	24	+	+	-	-	-	-	+	+++	+	+	+	-	-	+	-	-	-	-	-	-	-
28	25	++	+	+	+	+	+	+	++	-	+	+	-	+	+	-	-	-	-	-	-	-
	26	+	-	+	+	-	-	+	+	+	++	+	-	-	+	-	-	-	-	-	-	-
	27	+	+	-	-	-	-	+	-	+	+	+	+	-	+	-	-	-	-	-	-	-

^a^no detected;+:-10^4^ copies/mg; ++:10^4^ copies/mg;+++:10^5^- copies/mg

^b^copies/μL

## Discussion

Due to the lack of a cell culture system for propagating DuCV, many features of the genomic structure, function and molecular biology of DuCV remain unknown, even though DuCV was identified more than ten years ago. In particular, the molecular basis of the pathogenesis of this virus is unclear.

Reverse genetics is a powerful tool for addressing these questions, and in this study, we demonstrated the first generation of an infectious DNA clone of Sichuan isolate GH01. The main aim of our work was to produce a tandem-dimerized DuCV DNA clone. We further inserted an *Xho* I restriction enzyme site into the DuCV genome of the pIC-2DuCV clone to introduce a genetic marker that could be used to discriminate between the cloned virus and the potential indigenous viruses in the subsequent animal study. The recombinant pIC-2DuCV and pIC-Mu2DuCV plasmids and the pUC19 vector were inoculated intramuscularly into five ducklings. It appeared that both the pIC-2DuCV and pIC-Mu2DuCV DNA concatemers were replication-competent when transfected *in vivo* because they mimicked the natural DuCV circular genome. The rescue of pIC-2DuCV and pIC-Mu2DuCV was then demonstrated through *in vivo* animal experiments.

Ideally, the sequence of an infectious clone should be completely identical to that of the parental virus, but this goal is difficult to achieve in practice because during clone construction, some mutations will invariably occur during PCR amplification. In addition, other mutations have to be introduced for cloning purposes and for distinguishing the mutant from the parental virus. Thus, comparing the biological properties of an infectious clone-derived virus and its parental strain is important. Subsequent analysis revealed that the rescued and parental viruses had nearly identical biological characteristics in terms of clinical features, antigenicity, proliferation, distribution, and overall infectivity. The genetic marker that was engineered into the clone was used to confirm that the progeny virus was derived from the DNA clone and not from a contaminating virus. The number of copies of the engineered virus genome increased during infection, with the highest numbers (8.34 × 10^4^) observed at 21 DPC, and this increase was followed by a decline, which was similar to the trend observed for the parental virus (5.47 × 10^5^). The real-time qPCR results showed that the rescued virus displayed growth kinetics very similar to those of the parental virus. The titers of the parental virus were usually slightly higher than the those obtained for the rescued virus, a result that may be real and reflect mutations and genetic defects of the rescued virus, but the differences in titers and replications were quite small at all times. The virus that was rescued from ducklings infected with this mutated, full-length clone had the same growth properties as the parental virus, which demonstrates that the infectious DuCV clone is an excellent tool for site-directed mutagenesis and, importantly, that the recombinant virus generated in this study is capable of establishing an infection and displays similar pathogenicity as the parental virus in the natural host.

## Conclusion

In conclusion, we constructed a genetically stable and similarly pathogenic infectious clone of DuCV, which will facilitate studies of the pathogenesis, host tropism, replication, and transcription of this virus. This study shows that the rescued viruses are not significantly different but exhibit lower pathogenicity and proliferation ability compared with the parental virus. Further study is needed to explore the replication, host-DuCV interactions, and clinical significance of RMDV and WT-DuCV. In summary, we report the first demonstration that cloned DuCV genomic DNA with a genetic marker is infectious when directly injected into ducklings. In addition, we confirmed that the clinical signs of the ducklings injected with RMDV are similar to those of ducklings injected with WT-DuCV.
